# COGNITIVE PERFORMANCE AND COGNITIVE COMPLAINTS WITH ATTITUDES TOWARD AGING IN MIDLIFE AND OLD AGE

**DOI:** 10.1093/geroni/igz038.1414

**Published:** 2019-11-08

**Authors:** Jelena Siebert, Jelena S Siebert, Tina Braun, Hans-Werner Wahl

**Affiliations:** 1 Heidelberg University, Heidelberg, Germany; 2 Heidelberg University, Heidelberg, Baden-Wurttemberg, Germany; 3 Universität der Bundeswehr, Neubibberg, Bayern, Germany

## Abstract

Converging longitudinal research suggests that more negative views on aging go along with higher cognitive impairment. In some contrast, although conceptually suggested, possible reciprocal relationships remain less clear empirically. Using 20-year data from the Interdisciplinary Longitudinal Study of Adult Development (ILSE), we aim to better understand developmental co-dynamics between cognitive factors and attitude toward own aging (ATOA). Drawing on 1002 baseline participants (445 at T4) from two age cohorts (midlife: 40 years at baseline; old age: 60 years), longitudinal trajectories between ATOA, performance-based cognitive measures and subjective cognitive complaints are examined. Findings based on multi-group latent growth curve models (a) reveal substantial associations between ATOA and subjective complaints as well as objective cognition in both age groups; (b) confirm previous findings that ATOA predicts cognitive change over 20 years; and (c) find cognitive complaints but not cognitive performance able to predict change in ATOA in later life.

